# Effects of Long-Term Use of Insoles with a Toe-Grip Bar on the Balance, Walking, and Running of Preschool Children: A Randomized Controlled Trial

**DOI:** 10.1155/2020/1940954

**Published:** 2020-04-03

**Authors:** Hideki Nakano, Shin Murata, Teppei Abiko, Nozomi Mitsumaru, Atsuko Kubo, Mizuki Hachiya, Dai Matsuo, Michio Kawaguchi

**Affiliations:** ^1^Department of Physical Therapy, Faculty of Health Sciences, Kyoto Tachibana University, 34 Yamada-cho, Oyake, Yamashina-ku, Kyoto-shi, Kyoto-fu 607-8175, Japan; ^2^Department of Rehabilitation Sciences, Faculty of Rehabilitation Sciences, Nishikyushu University, 4490-9 Ozaki, Kanzaki-machi, Kanzaki-shi, Saga-ken 842-8585, Japan; ^3^ASICS Trading Company Limited, 3-5-2 Yasakadai, Suma-ku, Kobe-shi, Hyogo-ken 654-0161, Japan

## Abstract

This randomized controlled study is aimed at investigating the effects of long-term use of insoles with a toe-grip bar on the balance, walking, and running of preschool children. Fifty-two preschool children were randomly assigned to an intervention group or control group. Children included in the intervention group wore shoes with insoles that had a toe-grip bar, and those in the control group wore shoes with regular insoles without a toe-grip bar for 4 weeks while they were at school. The center of gravity sway (total trajectory length and envelope area), walking parameters (walking speed, cadence, stride length, step length, stance time, and swing time), and time to run 25 m were measured before and after the intervention. The 25 m running time of the intervention group was significantly improved after the intervention (*F* = 5.66; *p* < 0.05). This study suggests that insoles with a toe-grip bar may contribute to improvements in the running of preschool children.

## 1. Introduction

In the standard human standing position, the foot is the only body part that is in contact with the ground. Movable toes play an important role in posture control, walking, and running [1, 2]. Previous studies have shown that toe-grip force, the so-called toe-grip strength or toe flexor strength, decreases with age [3–6] and that decreased toe-grip strength is associated with problems with balance and walking ability and the occurrence of falls in the elderly [7–11]. Decreased toe-grip strength has also been observed in children and is particularly related to floating toes and flat feet [12, 13]. Furthermore, it has been reported that the toe-grip strength of children is related to lower limb physical activities, such as running [14, 15]. Therefore, it can be deduced that improving the toe-grip strength is important for maintaining and improving the health of children and the elderly.

Studies have shown that progressive resistance training and toe-grip-related training, such as performing towel-gathering exercises, are effective methods for improving the toe-grip strength [16, 17]. However, if this training is not performed regularly, then it is difficult to maintain the beneficial effects. As a solution to this problem, insoles with a toe-grip bar have been developed recently that improve the toe-grip strength while the individual is walking [18, 19]. According to previous studies, the toe-grip bar, which is a convex structure, is placed at the central part of the proximal phalanx from the first to the fifth toe. During the terminal stance phase of walking, the toes perceive the toe-grip bar and the reflex toe-grip movement is increased. The toe-grip movement occurs subconsciously when the individual walks, resulting in improved toe-grip strength [18, 19]. It has been reported that the toe-grip strength and toe flexibility of healthy individuals who wore insoles with a toe-grip bar experienced significant improvement after the intervention compared to healthy individuals who wore general insoles [18]. Moreover, it was reported that the toe-grip strength and body sway of the elderly significantly improved after the intervention phase that involved insoles with a toe-grip bar compared with after the baseline phase that involved general insoles [19]. Therefore, we hypothesized that insoles with a toe-grip bar may improve the physical performance of the lower limbs, including balance, walking, and running, of children. This study is aimed at investigating the effects of long-term use of insoles with a toe-grip bar on the balance, walking, and running of preschool children.

## 2. Materials and Methods

### 2.1. Participants

Fifty-two preschool children (mean age ± standard deviation (SD): 67.50 ± 3.83 months; 18 boys, 34 girls) participated in this study. Children were excluded if they had orthopedic, neurological, or psychiatric diseases that might influence the results. In this study, normal children who did not have any foot problems were included because it was a preliminary study of normal children that was performed before studying children with foot problems such as reduced toe-grip strength, floating toes, and flat feet.

The study was conducted according to the principles of the Declaration of Helsinki and was approved by the local Institutional Ethics Committee (Kyoto Tachibana University). All guardians provided their written informed consent, and the participants were free to withdraw from the study at any time.

### 2.2. Procedures

**Figure 1:**
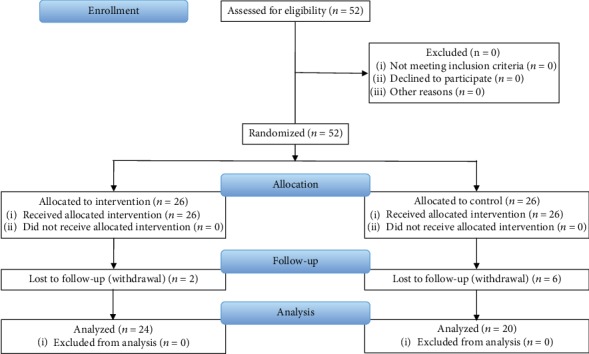
CONSORT flow chart.

Figure 1 shows the CONSORT flow chart. Participants were randomly assigned to an intervention group (*n* = 26; 9 boys, 17 girls) or control group (*n* = 26; 9 boys, 17 girls) using random numbers generated by Microsoft Excel 2010 (Microsoft, Redmond, WA, USA). Randomization was conducted by the study investigators. All participants were blinded to the group in which they were included.

**Figure 2:**
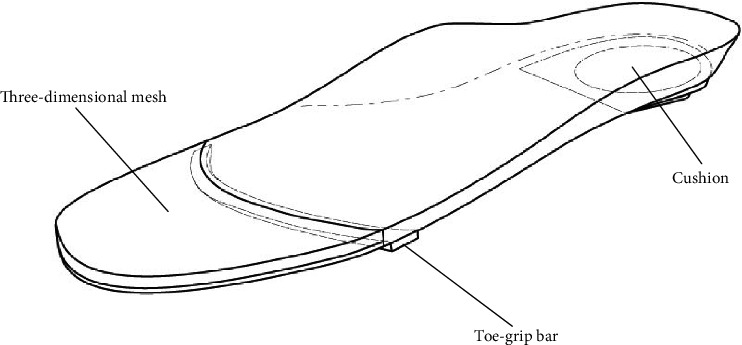
The insoles used in this study consisted of a toe-grip bar and a three-dimensional mesh.

The intervention group wore shoes with insoles with a toe-grip bar [18, 19]. The mid and rear parts of the insole were made of synthetic resin foam, and the toe section was made from synthetic fiber with high repulsion properties (known as three-dimensional mesh). The toe-grip bar was placed at the central part of the proximal phalanx from the first to the fifth toe (Figure 2). During the terminal stance phase of walking, the toes perceived the toe-grip bar and the reflex toe-grip movement was increased. The toe-grip movement occurred subconsciously while the participant was walking, resulting in improved toe-grip strength [18, 19].

The control group wore shoes with regular insoles without the toe-grip bar or a toe section made from synthetic fiber with high repulsion properties. The structure of the insoles was the same as that of the insoles used by the intervention group. Except for the insoles, both groups wore the same standard shoes provided by the study investigators. Therefore, the movements of the foot and ankle were almost the same in both groups, and only the movements of the toes were adjusted to be different. Both groups wore the shoes 5 times per week for 4 weeks while at school.

### 2.3. Measures

The center of gravity sway (total trajectory length (TL) and envelope area (EA)), walking parameters (walking speed, cadence, stride length, stance time, and swing time), and 25 m running time were measured before and immediately after the 4-week intervention.

The center of gravity sway was measured using a stabilometer (GP-7; Anima Co., Ltd., Tokyo, Japan). The participants were instructed to stand in a two-leg stance under the following standardized conditions: barefoot, eyes open, looking at a target placed on a wall 2 m away at eye level, and arms at the sides [18, 19]. Data were collected after the participants had stood for 5 s to exclude the influence of initial sway. Data were measured for 10 s at a sampling rate of 20 Hz [20]. The mean TL and EA values of the center of pressure were calculated based on two measurements.

The walking parameters were assessed using a WalkWay device (WalkWay MW-1000; Anima Co., Ltd., Tokyo, Japan) [21]. This device calculates temporal and spatial gait parameters from the distribution of foot pressure and consists of a 2,400 × 800 × 5 mm (length × width × thickness) sheet, a sensor with spatial resolution of 10 × 10 mm, and 14,400 measurement points. Gait consisted of the following sections: 2 m acceleration, 2.4 m measurement, and 2 m deceleration (total, 6.4 m). The data were measured at a sampling rate of 100 Hz. Participants were instructed to walk barefoot at normal speed. The measurement was performed twice, and the average values of the walking speed (cm/s), cadence (steps/min), stride length (cm), step length (cm), stance time (s), and swing time (s) were calculated.

The 25 m running time was measured using a digital stopwatch [22, 23]. The run began from a standing start. A starter gave the following instructions: “ready” and “go.” The participants started to run when they heard “go.” The amount of time required to reach the goal of 25 m was measured.

### 2.4. Statistical Analyses

The baseline characteristics of the intervention and control groups were compared to determine if the two groups were comparable. The Kolmogorov–Smirnov test was used to test the normality of distributions. Differences between groups were analyzed using Student's *t*-tests for normally distributed variables and the Mann–Whitney *U* test for variables that were not normally distributed. Measurement items were analyzed using a two-way repeated measures analysis of variance (ANOVA). Post hoc Bonferroni testing was used to assess the group or time period that showed significant differences. Statistical analyses were performed with SPSS 24.0 (IBM, Chicago, IL, USA). The level of significance was <5%.

## 3. Results

Two participants from the intervention group and six participants from the control group who could not be assessed after training were excluded. Before training, there were no significant differences between the groups regarding age, height, body weight, TL, EA, walking speed, cadence, stride length, step length, stance time, swing time, and 25 m running time (all *p* > 0.05) (Table 1). Two-way repeated measures ANOVA showed that the 25 m running time was mainly affected (*F* = 5.66; *p* < 0.05). Post hoc Bonferroni comparisons revealed that the 25 m running time significantly improved in the intervention group after training (*p* < 0.05) (Table 2).

## 4. Discussion

The present study suggests that long-term use of insoles with a toe-grip bar may contribute to improvements in running among preschool children. Our previous studies reported that the continuous use of insoles with a toe-grip bar for 4 weeks increased the toe-grip strength of young adults and older individuals [18, 19]. The results of this study similarly suggest that continuous use of insoles with a toe-grip bar may enhance the toe-grip strength of preschool children. A previous study reported that toes can generate propulsive force during running [1]. Moreover, a significant association between toe-grip strength and 50 m running time of elementary school children was reported [14]. Additionally, a significant association between toe-grip strength and 25 m running time of preschool children was reported [15]. Hence, it may be inferred that the increased toe-grip strength improved the 25 m running time of preschool children in this study.

Our previous studies reported an improvement in the body sway of older adults with poor balancing ability after they wore insoles with a toe-grip bar [19]; however, this was not observed in younger adults with normal balancing ability [18]. Therefore, the effects of insoles with a toe-grip bar may be observed in individuals with reduced physical performance of the lower limbs or during situations requiring dynamic performance and maximum muscle strength output of the toe grip. In this study, no significant differences were observed in the center of gravity sway; it did not decrease in the preschool children. Furthermore, it was found that the normal walking parameters do not require maximum muscle strength output from preschool children.

There were some limitations to this study. First, the number of steps during the training period was not measured. Therefore, this study could not exclude the influence of steps during the training period. Further studies are necessary to measure the steps during the training period. Second, it is unclear how long the beneficial effects obtained using the insole could be maintained. Further studies are needed to investigate the sustainability of the benefits obtained by using insoles. Third, this study did not include measurements of the toe-grip strength. Therefore, we do not know the extent of increase of the toe-grip strength after the intervention. Hence, future studies should clarify the effects of toe-grip bar on the toe-grip strength of preschool children.

## 5. Conclusions

This study investigated the effects of long-term use of insoles with a toe-grip bar on the balance, walking, and running of preschool children. The 25 m running time of the intervention group showed significant improvements after the intervention. The results of this study suggest that insoles with a toe-grip bar may contribute to an improvement in running for preschool children. Moreover, insoles with a toe-grip bar might help prevent floating toes and flat feet observed in children. Furthermore, they may be an important tool for maintaining and improving the health of children. Additional studies should be performed to clarify their clinical relevance for children with foot problems based on the findings of this study.

## Figures and Tables

**Table 1:** Characteristics of the intervention and control groups.

Parameters	Intervention group		Control group		*p* value
	Mean	SD	Mean	SD	
Age (months)	67.63	4.00	67.35	3.72	0.82
Height (cm)	110.95	5.16	110.94	4.38	1.00
Body weight (kg)	18.92	2.00	18.65	1.93	0.66
Total trajectory length (cm)	19.36	6.71	21.06	5.52	0.37
Envelope area (cm^2^)	1.08	0.42	1.36	0.83	0.17
Walking speed (cm/s)	111.70	26.72	114.26	12.04	0.68
Cadence (steps/min)	145.40	23.28	145.25	12.60	0.98
Stride length (cm)	91.69	11.59	95.08	8.47	0.28
Stance time (s)	0.48	0.09	0.48	0.04	0.72
Swing time (s)	0.36	0.04	0.36	0.03	0.94
25 m running time (s)	6.72	0.53	6.49	0.67	0.20

SD: standard deviation.

**Table 2:** Comparison of parameters before and after training of the intervention and control groups.

Parameters	Group	Before		After		Main effect of time	
		Mean	SD	Mean	SD	*F*-value	*p* value
Total trajectory length (cm)	Intervention	19.36	6.71	17.57	4.74	2.18	0.15
	Control	21.06	5.52	20.64	5.68		
Envelope area (cm^2^)	Intervention	1.08	0.42	1.08	0.64	0.02	0.88
	Control	1.36	0.83	1.33	0.53		
Walking speed (cm/s)	Intervention	111.70	26.72	113.46	22.96	0.63	0.43
	Control	114.26	12.04	108.01	17.77		
Cadence (steps/min)	Intervention	145.40	23.28	143.53	19.33	3.12	0.08
	Control	145.25	12.60	138.74	14.72		
Stride length (cm)	Intervention	91.69	11.59	94.77	13.50	0.11	0.74
	Control	95.08	8.47	93.28	12.06		
Stance time (s)	Intervention	0.48	0.09	0.48	0.08	2.96	0.09
	Control	0.48	0.04	0.50	0.06		
Swing time (s)	Intervention	0.36	0.04	0.37	0.04	1.04	0.31
	Control	0.36	0.03	0.37	0.04		
25 m running time (s)	Intervention	6.72	0.53	6.46	0.58	5.66	0.02^∗^
	Control	6.49	0.67	6.37	0.64		

SD: standard deviation; ^∗^*p* < 0.05: significant difference before and after the intervention.

## Data Availability

The data used to support the findings of this study are available from the corresponding author upon request.

## References

[1] Mann R. A., Hagy J. L. (1979). The function of the toes in walking, jogging and running. *Clinical Orthopaedics and Related Research*.

[2] Saeki J., Tojima M., Torii S. (2015). Clarification of functional differences between the hallux and lesser toes during the single leg stance: immediate effects of conditioning contraction of the toe plantar flexion muscles. *Journal of Physical Therapy Science*.

[3] Endo M., Ashton-Miller J. A., Alexander N. B. (2002). Effects of age and gender on toe flexor muscle strength. *The Journals of Gerontology. Series A, Biological Sciences and Medical Sciences*.

[4] Menz H. B., Zammit G. V., Munteanu S. E., Scott G. (2006). Plantarflexion strength of the toes: age and gender differences and evaluation of a clinical screening test. *Foot & Ankle International*.

[5] Uritani D., Fukumoto T., Matsumoto D., Shima M. (2014). Reference values for toe grip strength among Japanese adults aged 20 to 79 years: a cross-sectional study. *Journal of Foot and Ankle Research*.

[6] Mickle K. J., Angin S., Crofts G., Nester C. J. (2016). Effects of age on strength and morphology of toe flexor muscles. *The Journal of Orthopaedic and Sports Physical Therapy*.

[7] Menz H. B., Morris M. E., Lord S. R. (2005). Foot and ankle characteristics associated with impaired balance and functional ability in older people. *The Journals of Gerontology. Series A, Biological Sciences and Medical Sciences*.

[8] Misu S., Doi T., Asai T. (2014). Association between toe flexor strength and spatiotemporal gait parameters in community-dwelling older people. *Journal of Neuroengineering and Rehabilitation*.

[9] Menz H. B., Morris M. E., Lord S. R. (2006). Foot and ankle risk factors for falls in older people: a prospective study. *The Journals of Gerontology. Series A, Biological Sciences and Medical Sciences*.

[10] Mickle K. J., Munro B. J., Lord S. R., Menz H. B., Steele J. R. (2009). ISB Clinical Biomechanics Award 2009: toe weakness and deformity increase the risk of falls in older people. *Clinical Biomechanics*.

[11] Tsuyuguchi R., Kurose S., Seto T. (2018). Toe grip strength in middle-aged individuals as a risk factor for falls. *The Journal of Sports Medicine and Physical Fitness*.

[12] Tasaka S., Matsubara K., Nishiguchi S. (2016). Association between floating toe and toe grip strength in school age children: a cross-sectional study. *Journal of Physical Therapy Science*.

[13] Tashiro Y., Fukumoto T., Uritani D. (2015). Children with flat feet have weaker toe grip strength than those having a normal arch. *Journal of Physical Therapy Science*.

[14] Morita N., Yamauchi J., Kurihara T. (2015). Toe flexor strength and foot arch height in children. *Medicine and Science in Sports and Exercise*.

[15] Uritani D., Fukumoto T., Matsumoto D., Shima M. (2017). Association between toe grip strength and physical performance among Japanese preschool children. *Clinicl Reserch on Foot & Ankle*.

[16] Mickle K. J., Caputi P., Potter J. M., Steele J. R. (2016). Efficacy of a progressive resistance exercise program to increase toe flexor strength in older people. *Clinical Biomechanics*.

[17] Tsuyuguchi R., Kurose S., Seto T. (2019). The effects of toe grip training on physical performance and cognitive function of nursing home residents. *Journal of Physiological Anthropology*.

[18] Nakano H., Murata S., Abiko T. (2017). Effect of insoles with a toe-grip bar on toe function and standing balance in healthy young women: a randomized controlled trial. *Rehabilitation Research and Practice*.

[19] Nakano H., Murata S., Abiko T. (2018). Effect of insoles with a toe-grip bar on toe-grip strength and body sway in middle-aged and elderly women. *Topics in Geriatric Rehabilitation*.

[20] Rival C., Ceyte H., Olivier I. (2005). Developmental changes of static standing balance in children. *Neuroscience Letters*.

[21] Yozu A., Hamada M., Sasaki T., Tokushige S., Tsuji S., Haga N. (2017). Development of a novel system to quantify the spatial–temporal parameters for crutch-assisted quadrupedal gait. *Advanced Robotics*.

[22] Nguyen T., Obeid J., Timmons B. W. (2011). Reliability of fitness measures in 3- to 5-year-old children. *Pediatric Exercise Science*.

[23] Hirao A., Murata S., Kubo A., Hachiya M., Mitsumaru N., Asami T. (2015). Association between occlusal force and physical functions in preschool children: a comparison of males and females. *Journal of Physical Therapy Science*.

